# Cardiac Effects of a Single Dose of Pimobendan in Cats With Hypertrophic Cardiomyopathy; A Randomized, Placebo-Controlled, Crossover Study

**DOI:** 10.3389/fvets.2019.00015

**Published:** 2019-02-04

**Authors:** Maureen S. Oldach, Yu Ueda, Eric S. Ontiveros, Samantha L. Fousse, Samantha P. Harris, Joshua A. Stern

**Affiliations:** ^1^William R. Prichard Veterinary Medical Teaching Hospital, University of California, Davis, Davis, CA, United States; ^2^Department of Medicine and Epidemiology, School of Veterinary Medicine, University of California, Davis, Davis, CA, United States; ^3^Department of Cellular and Molecular Medicine, Sarver Heart Center, College of Medicine, University of Arizona, Tucson, AZ, United States

**Keywords:** feline, vetmedin, pharmacodynamics, HCM, obstruction, outflow, safety

## Abstract

**Background:** Pimobendan has been shown to impart a significant survival benefit in cardiomyopathic cats who receive it as part of heart failure therapy. However, use of pimobendan remains controversial in cats with hypertrophic cardiomyopathy (HCM) due to lack of pharmacodynamic data for pimobendan in cats with HCM and due to theoretical concerns for exacerbating left ventricular outflow tract obstructions.

**Hypothesis/Objectives:** Our objective was to investigate the cardiac effects of pimobendan in cats with HCM. We hypothesized that pimobendan would not exacerbate left ventricular outflow tract obstructions and that it would improve echocardiographic measures of diastolic function.

**Animals:** Thirteen purpose-bred cats were studied from a research colony with naturally-occurring HCM due to a variant in myosin binding protein C.

**Methods:** Cats underwent two examinations 24 h apart with complete standard echocardiography. On their first day of evaluation, they were randomized to receive oral placebo or 1.25 mg pimobendan 1 h prior to exam. On their second examination, they were crossed over and received the remaining treatment. Investigators were blinded to all treatments.

**Results:** The pimobendan group had a significant increase in left atrial fractional shortening (pimobendan group 41.7% ± 5.9; placebo group 36.1% ± 6.0; *p* = 0.04). There was no significant difference in left ventricular outflow tract (LVOT) velocities between the groups (pimobendan group 2.8 m/s ± 0.8; placebo group 2.6 m/s ± 1.0). There were no significant differences between the number of cats with LVOT obstructions between groups (12 in pimobendan group; 11 in placebo group; *p* = 1.00). There were no detectable differences in any systolic measures, including left ventricular fractional shortening, mitral annular plane systolic excursion, and tricuspid annular plane systolic excursion. Doppler-based diastolic function assessment was precluded by persistent tachycardia.

**Conclusions:** Improved left atrial function in the pimobendan group could explain some of the reported survival benefit for HCM cats in CHF. Pimobendan did not exacerbate LVOT obstructions and thus may not be contraindicated in HCM cats with LVOT obstructions. Future studies are needed to better characterize other physiologic effects, particularly regarding diastolic function assessment, and to better assess safety of pimobendan over a longer time-course.

## Introduction

Pimobendan is a benzimidazole pyridazinone drug that has shown tremendous benefit in treatment of both canine congestive heart failure (CHF), pre-clinical dilated cardiomyopathy and preclinical degenerative valve disease (1–6). Its effects through calcium sensitization of the cardiac troponin C molecule and phosphodiesterase III inhibition impart increased inotropy and balanced vasodilation, leading to improved cardiac output with greater potency and without documented pro-arrhythmic risk posed by other inotropic agents, such as digoxin. Its effects have engendered the term inodilator to describe its actions (1).

The positive effects in prolonging survival times, improving quality of life, and prolonging time to the development of CHF in dogs is unequivocal (2–6). However, this benefit is not universally accepted or characterized in feline cardiac disease. Hypertrophic cardiomyopathy (HCM) is the most common cardiac disease in cats, and affects 14.5–14.7% of the domestic cat population (7, 8). HCM is characterized by concentric hypertrophy of the left ventricular myocardium and concurrent fibrosis and stiffening of the heart with a preserved or hyperdynamic systolic function. The predominant etiology of cardiac decompensation in these patients is diastolic dysfunction. As a complicating factor, 47.5–67% of HCM cats presenting to referral hospitals have concurrent left ventricular outflow tract obstructions (LVOTO) (9, 10). These obstructions can be secondary to several etiologies including systolic anterior motion of the anterior mitral leaflet (SAM), asymmetric septal hypertrophy, or a dynamic mid-ventricular obstruction lesion caused by mid-ventricular and/or papillary muscle hypertrophy. Cats and humans with HCM and concurrent LVOTO are termed hypertrophic obstructive cardiomyopathy. Hypertrophic obstructive cardiomyopathy is reported as a main cause of symptoms of angina and syncope in humans with this disease (11, 12). There is concern that a positive inotrope and afterload reducing drug, such as pimobendan, could exacerbate or induce these dynamic obstructions in cats with HCM, which could cause hypotension, syncope, and exercise intolerance. As such, some have described pimobendan as contraindicated for use in animals with HCM despite the positive clinical outcomes in the literature (13, 14).

Cats with HCM have a 28.3% risk of developing CHF, making heart failure therapy an important part of HCM disease management (15). In a previous retrospective case-control study, pimobendan given to cats with HCM and CHF provided a significant survival benefit of 523 days over cats who did not receive pimobendan as a part of their treatment (14). The etiology of this survival benefit remains unknown. Beyond the inodilator properties of pimobendan, additional pharmacodynamic effects have been reported in cats and other species. Pimobendan improves diastolic function in dogs with pacing-induced cardiomyopathy and humans with idiopathic dilated cardiomyopathy (16, 17). A similar inodilator drug, levosimendan, has also been shown to have positive lusitropic effects perioperatively in humans with preserved ejection fraction who were receiving aortic valve replacements and coronary artery bypass grafts (18, 19). Pimobendan has also been shown to decrease left atrial (LA) size in normal cats, implying some lusitropic or LA function benefit (20). These pharmacodynamic qualities of the drug may represent the source of benefit for cats with HCM and CHF, but to date, although many studies have documented that pimobendan is well-tolerated in cats, there have been no studies evaluating the pharmacodynamics of pimobendan in cats with HCM (14, 21–26). Thus, it remains unknown whether pimobendan could induce or exacerbate dynamic LVOTOs, and whether any positive effects can be documented in cats with HCM. This question translates to similar questions in human cardiac disease, where the inodilator, levosimandan has been shown to improve diastolic function in patients with heart failure with preserved ejection fraction (18, 19). As cats with HCM have recently been identified as an animal model for heart failure with preserved ejection fraction, elucidation of possible benefits in cats with HCM may also have implications for similar cardiac diseases in humans (9, 27, 28).

We sought to assess the acute pharmacodynamic effects of a single oral dose of pimobendan in cats with HCM using echocardiography, with a specific aim to investigate whether pimobendan could induce or exacerbate LVOTOs in this population. We hypothesized that pimobendan would not exacerbate LVOTOs. We also hypothesized that pimobendan would have positive lusitropic effects, as measured by echocardiography.

## Materials and Methods

### Animals

All animal procedures were in accordance with the National Research Council Guide for the Care and Use of Laboratory Animals using protocols approved by the Institutional Animal Care and Use Committee at the University of California, Davis. Thirteen purpose-bred mixed-breed cats were used from a research colony generated from a single Maine Coon/mixed-breed founder cat with naturally occurring HCM due to a variant in the myosin binding protein C gene (*MYBPC*). Six of 13 cats were homozygous for the reported A31P *MYBPC3* variant, while 5 were heterozygous for this variant and 2 wild type for this variant and thus of unknown genetic etiology. Cats were identified as affected by HCM if there was segmental or diffuse interventricular or left ventricular posterior wall thickness exceeding 6 mm in the absence of hypertension or hyperthyroidism on at least two serial examinations >1 month apart, as identified on two-dimensional (2D) exam in the right parasternal short-axis or long-axis view.

### Experimental Design

The study was a randomized, double-blinded, cross-over design. Lactulose powder (placebo) and 1.25 mg pimobendan tablets were placed into identical opaque capsules to facilitate investigator blinding. The medications were randomized to group A or B by a single unblinded individual not involved in data acquisition or analysis. On their first evaluation (exam 1), cats were randomized by coin-flip to receive either medication A or B. One capsule of the assigned medication was administered orally; 30 min following the administration, cats were sedated with acepromazine (0.5 mg) and butorphanol (1 mg) administered intramuscularly in the epaxial muscles. One hour after study medication administration, echocardiography was performed. This time-frame was chosen based on a prior pharmacokinetics study in cats, showing a predicted time to peak plasma concentration of 54 min (22). The cats were then monitored for adverse effects, defined as any adverse event occurring in temporal proximity to administration of the study drug, including collapse, vomiting, diarrhea, agitation, ptyalism, and arrhythmias. Arrhythmias were monitored for during the echocardiogram by a lead II electrocardiographic rhythm strip. After full recovery, the cats were returned to their colony housing. At least 24 h after their initial examination, they were presented for the crossover portion of the study, during which the same procedure described above was performed with administration of the opposite medication (i.e., cats who received medication B during exam 1 received medication A on exam 2). Twenty-four hours was deemed a sufficient wash-out period given that it was >5 times the reported elimination half-life of pimobendan (1.3 h) (22).

### Echocardiography

Thirty minutes after sedation with 1.0 mg butorphanol intramuscularly and 0.5 mg acepromazine intramuscularly, cats underwent echocardiographic exams. All echocardiographic examinations were performed by a single investigator (MSO) on a Phillips EPIQ CVx ultrasound machine using a 12 MHz transducer with harmonics. Complete 2D, motion mode (m-mode), color, and spectral Doppler echocardiography was performed from standard imaging planes in right and left lateral recumbency, as previously described (29); a concurrent lead II continuous ECG was also recorded and monitored for arrhythmias. Images were stored and measurements were performed by a single investigator (MSO) at a remote workstation using Siemens Syngo Dynamics proprietary software.

Assessment of left ventricle (LV) hypertrophy was made from 2D examination in right parasternal short axis or long axis imaging using an inner edge to inner edge measurement technique; segmental or diffuse interventricular or left ventricular posterior wall thickness exceeding 6 mm in the absence of hypertension or hyperthyroidism on at least two serial examinations >1 month apart was considered consistent with HCM. Dynamic right ventricular outflow tract obstruction (DRVOTO) was identified from the right parasternal short axis view, and was defined as the presence of color Doppler turbulence within the proximal infundibular region of the right ventricular outflow tract (RVOT), with a spectral continuous wave Doppler velocity >1.6 m/s, in the absence of other cardiac pathology (i.e., cardiac shunting lesions or pulmonic stenosis, as this velocity exceeds the upper limits of normal RVOT velocity (30). LVOTO was identified from the left parasternal 5-chamber view, and was defined as the presence of color Doppler flow turbulence in the left ventricular outflow tract (LVOT) and a late-peaking spectral continuous wave Doppler signal with a velocity of >1.9 m/s, the upper end of normal LVOT velocity (30). The cursor was aligned with the color Doppler turbulent flow, and the maximal modal velocity obtained was recorded. Mid LVOTO was identified when there was mid-ventricular hypertrophy with color flow turbulence arising in the mid LV during systole in combination with the spectral Doppler features mentioned previously. The presence or absence of SAM was also noted based on 2D exam.

Additional echo measurements were acquired as follows: left atrial fractional shortening (LAFS) with m-mode in the right parasternal short axis view (31); LV fractional shortening with m-mode from the right parasternal short axis view; 2D left atrial to aortic ratio from the right parasternal short axis view; the maximal left atrial diameter in 2D from the right parasternal long-axis view; pulsed wave tissue Doppler imaging of the lateral mitral annulus S' wave from the left apical 4-chamber view; m-mode mitral annular plane systolic excursion of the septal annulus from the left apical 4-chamber view; LV isovolumetric relaxation time using spectral pulsed wave Doppler from the left parasternal 4-chamber view; tricuspid annular plane systolic excursion from the left parasternal apical 4-chamber view optimized for the RV; spectral pulsed wave Doppler left auricular flow velocity from the left parasternal cranial long axis view optimized for the left auricle. Transmitral spectral Doppler and diastolic tissue Doppler imaging was attempted from the left apical 4-chamber view, but was unsuccessful due to persistent tachycardia resulting in fusion of the early and late filling waves.

### Statistics

Statistical analysis was performed using GraphPad Prism version 7.0 and GraphPad Quickcalcs. (GraphPadSoftware Inc., La Jolla, CA, USA). All variables were first analyzed for column statistics and tested for normality by visual inspection and D'Agostino Pearson Omnibus Normality test. Normally distributed data are reported as mean ± standard deviation. Non-parametric data are reported as median and interquartile range. Group A vs. group B statistical comparisons were performed for paired data using a paired *t*-test (when normally distributed) and a Wilcoxon signed-rank matched pairs test (when non-parametric). Categorical variables were coded as 0 (no) and 1 (yes) to create a 2 × 2 contingency table for the purposes of statistical analysis. These variables were tested for significant differences between groups A and B using a Fisher's exact test. Correlations between LVOT maximal velocity and heart rate (HR) at the time of measured velocity as well as RVOT maximal velocity and HR at the time of measured velocity were evaluated using a Pearson Correlation Coefficient. A *p* < 0.05 was considered significant. When values were missing on some cats, the corresponding value in the cross-over group was deleted to permit continuation of paired sample testing. In the case where values were unavailable, the number of animals included in the paired sample analysis is reported (n = x).

Categorical variables were coded as 0 (no) and 1 (yes) to create a 2 × 2 contingency table for the purposes of statistical analysis. These variables were tested for significant differences between groups A and B using a Fisher's exact test through Graphpad QuickCalcs (GraphPadSoftware Inc., La Jolla, CA, USA).

## Results

Thirteen purpose-bred mixed-breed cats were included in the study. Median age of the cats was 50 months (range 12–83 months). All cats had a maximal segmental or uniform wall thickness of >6 mm in both treatment groups. All cats had systolic heart murmurs at both examinations, with a median murmur grade of IV/VI in both treatment groups and a range of II-IV/VI. Murmur grades were not significantly different between groups (*p* > 0.9999). There was no significant difference in HR, LA size, and segmental wall thickness between the two treatment groups (Table 1).

**Table 1:** Treatment group phenotypic comparisons.

	**Placebo treatment group**	**Pimobendan treatment group**	***P*-value**
HR (mean bpm ± SD)	226 ± 31	231 ± 26	0.65
Thickest wall segment [median mm (IQR)]	6.9 (6.6–7.1)	6.9 (6.7–7.1)	0.51
LAlax [median mm (IQR)]	1.26 (1.2–1.4)	1.2 (1.1–1.6)	0.27

*HR, heart rate; bpm, beats per minute; SD, standard deviation; IQR, interquartile range; LAlax, maximal left atrial diameter*.

**Figure 1:**
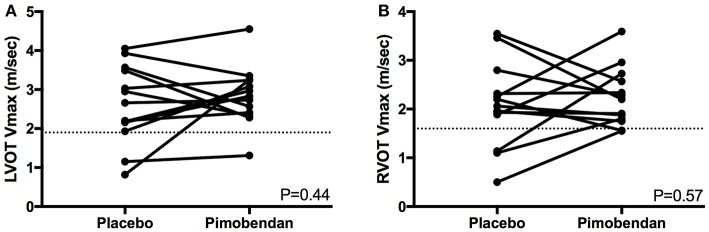
**(A)** A paired *t*-test comparison of maximal left ventricular outflow tract velocity (LVOT Vmax) (m/sec) between cats receiving placebo and pimobendan is shown. No significant differences were identified (*P* = 0.44). A dashed line represents the threshold considered to constitute left ventricular outflow tract obstruction in this study at 1.9 m/s. **(B)** A paired *t*-test comparison of maximal right ventricular outflow tract velocity (RVOT Vmax) (m/sec) between cats receiving placebo and pimobendan is shown. No significant differences were identified (*P* = 0.57). A dashed line represents the threshold considered to constitute right ventricular outflow tract obstruction in this study at 1.6 m/s.

There were no significant differences between the number of cats in each group with SAM, LVOTO, or mid-LVOTO (Table 2). Eleven cats in the placebo group had an LVOTO; 10 of these cats had a mid-LVOTO and 4 had SAM. Twelve cats in the pimobendan treatment group had LVOTO, with 11 experiencing mid-LVOTO and 5 with SAM. One cat without SAM in the placebo group had documented SAM in the pimobendan group; no cats without SAM in the pimobendan group had documented SAM in the placebo group. Ten cats in the placebo and 11 cats in the pimobendan group had DRVOTO; this number was not significantly different between groups (Table 2). The LVOT and RVOT velocities in the treatment and placebo group did not differ significantly (Table 3; Figure 1). The median LVOT velocity in the placebo group was 2.66 m/s with a range of 0.82–4.05 m/s; the median LVOT velocity in the treatment group was 2.84 m/s with a range of 1.31–4.55 m/s (Figure 1).

**Table 2:** Categorical variable analysis.

	**Placebo treatment group (n)**	**Pimobendan treatment group (n)**	**Pimobendan vs. placebo *P*-value**
LVOTO	11	12	1.00
SAM	4	5	1.00
Mid-LVOTO	10	11	1.00
DRVOTO	10	11	1.00
VPCs	3	3	1.00
SPCs	0	0	1.00
Adverse events	1	1	1.00
HCM	13	13	1.00

*LVOTO, Left ventricular outflow tract obstruction; SAM, systolic anterior motion; DRVOTO, dynamic right ventricular outflow tract obstruction; VPCs, ventricular premature complexes; SPCs, supraventricular premature complexes; HCM, Hypertrophic cardiomyopathy*.

**Table 3:** Functional echocardiographic variables.

	**Placebo treatment group**	**Pimobendan treatment group**	***P*-value**
LAFS M-mode (mean%, SD)	36.1 ± 6.0	41.7 ± 5.9	0.04
LVFS M-mode (mean %, SD)	65.0 ± 13.6	68.3 ± 11.3	0.35
Mitral TDI S' [median m/s (IQR)]	0.09 (0.08–0.11)	0.09 (0.08–0.10)	0.50
MAPSE (mean mm, SD)	4.2 ± 0.7	3.8 ± 0.6	0.17
Auricular flow (mean cm/s, SD)	90.6 ± 13.1	94.5 ± 12.7	0.44
TAPSE (mean mm, SD)	8.3 ± 1.6	7.6 ± 1.6	0.10
IVRT (mean ms, SD)	44.1 ± 11.8	46.2 ± 9.3	0.60
LVOT Vmax (mean m/s, SD)	2.6 ± 1.0	2.8 ± 0.8	0.44
RVOT Vmax (mean m/s, SD)	2.1 ± 0.9	2.2 ± 0.6	0.57

*LAFS, left atrial fractional shortening; m-mode, motion mode; SD, standard deviation; LVFS, Left ventricular fractional shortening,; TDI, tissue Doppler imaging; IQR, interquartile range; MAPSE, mitral annular plane systolic excursion; TAPSE, tricuspid annular plane systolic excursion, IVRT, isovolumetric relaxation time, LVOT Vmax, left ventricular outflow tract maximal velocity; RVOT Vmax, Right ventricular outflow tract maximal velocity*.

**Figure 2:**
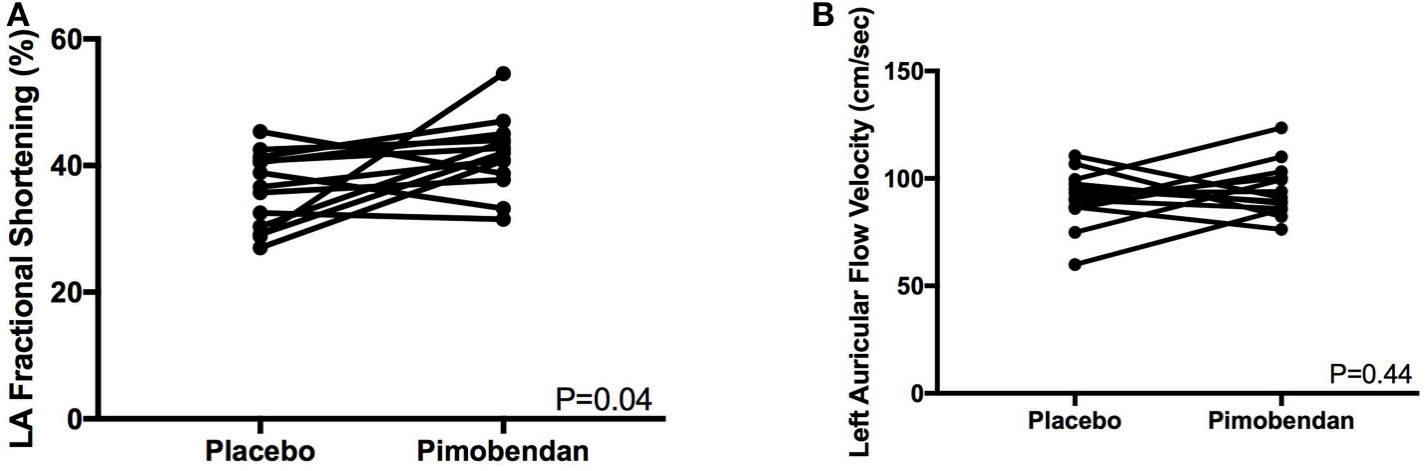
**(A)** A paired *t*-test comparison of left atrial (LA) fractional shortening (%) measured by m-mode between cats receiving placebo and pimobendan is shown. Cats receiving pimobendan had significantly increased left atrial fractional shortening (*P* = 0.04). **(B)** A paired *t*-test comparison of left auricular flow velocity (cm/sec) measured by spectral Doppler between cats receiving placebo and pimobendan is shown. No significant differences were identified (*P* = 0.44).

**Figure 3:**
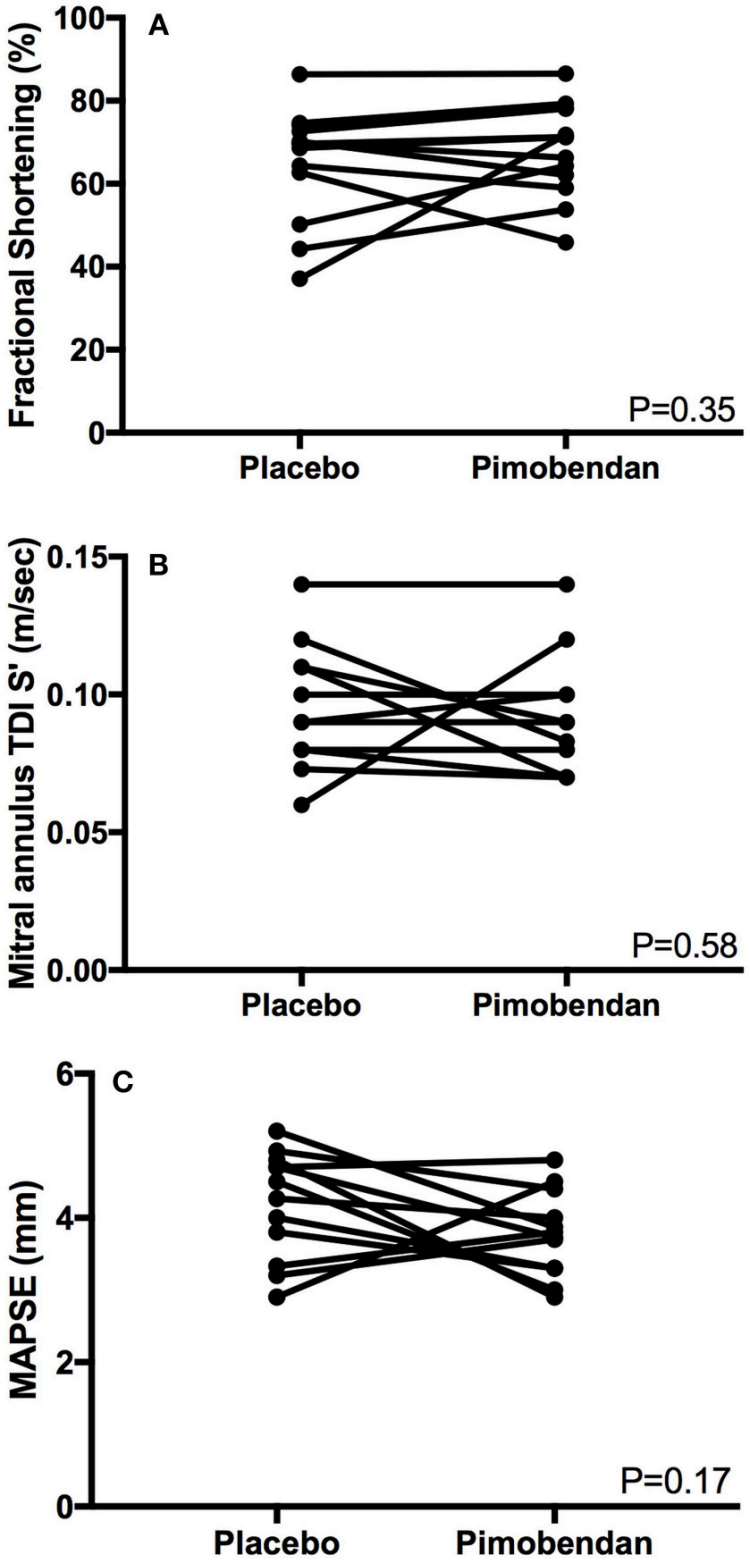
**(A)** A paired *t*-test comparison of left ventricular fractional shortening (%) measured by m-mode between cats receiving placebo and pimobendan is shown. No significant differences were identified (*P* = 0.35). **(B)** A Wilcoxon matched-pairs signed rank test comparison of lateral mitral annulus tissue Doppler imaging (TDI) S' wave (m/sec) between cats receiving placebo and pimobendan is shown. No significant differences were identified (*P* = 0.58). **(C)** A paired *t*-test comparison of septal mitral annular plane systolic excursion (MAPSE) (mm) measured by m-mode between cats receiving placebo and pimobendan is shown. No significant differences were identified (*P* = 0.17).

Functional echocardiographic values are presented in Table 3. Left atrial fractional shortening in the pimobendan treatment group was significantly greater than the placebo group (Figure 2A), however no differences in left auricular flow velocity were identified (Figure 2B). There were no significant differences in LV fractional shortening, mitral annulus tissue Doppler imaging S', or mitral annular plane systolic excursion M-mode (Figure 3).

No significant correlations were observed between LVOT maximal velocity and HR in the pimobendan group (*r* = 0.35, *p* = 0.24), or in the placebo group (*r* = 0.30 *p* = 0.32). No significant correlations were observed between RVOT maximal velocity and HR in the pimobendan group (*r* = −0.27, *p* = 0.37) or the placebo group (*r* = 0.49, *p* = 0.09).

There was no significant difference in the prevalence of ventricular arrhythmias between groups (Table 2). No supraventricular ectopy was noted in either group.

One cat from each group experienced an adverse event during the evaluation. There was no difference in the number of adverse events between groups (Table 2). One of these cats in the pimobendan group experienced transient bradycardia (125 bpm) and brief loss of postural tone during the examination. This patient demonstrated a notable stress response (HR > 300 bpm) despite sedation and this episode was considered likely pre-syncope. This cat did not experience a new obstruction with tachycardia. The placebo measured LVOT velocity for this cat was 4.05 m/s and was mildly increased to 4.55 m/s after pimobendan and this patient was observed to have both SAM and mid-LVOTO at both evaluations. Additionally, one cat in the placebo group experienced diarrhea upon the conclusion of the exam.

## Discussion

This study demonstrated that pimobendan has a positive effect on LA function, as shown by an increase in the LAFS m-mode, which was significantly increased compared with the placebo group. This finding could explain some of the survival benefit that has been documented in HCM cats with CHF receiving pimobendan (14). Prior studies have shown reduced LAFS in HCM cats with CHF, with these cats also having an increased risk for thromboembolic disease and death (31, 32). Similarly, an increased risk of thromboembolic disease in cats with reduced auricular flow velocities, another measure of LA function, has also been demonstrated (32). Improved LA function would improve LV filling and limit LA dilation, which may help prevent thromboembolic complications and improve CHF control.

It is interesting that although LAFS m-mode increased in the pimobendan group, the LV internal dimension in diastole did not increase, as an increase in LA emptying should improve LV filling and thus increase LV diastolic dimension. This discrepancy is most likely related to the challenges of accurate LV measurement in cats with tachycardia and irregular LV concentric hypertrophy. Persistent tachycardia likely limited imaging resolution, potentiating measurement error. In addition, many cats had asymmetric hypertrophy of their left ventricles, and identical imaging planes may not have been achieved between studies. Future clinical studies may benefit from selecting compliant patients where less significant tachycardia and better cooperation without the necessity for sedation may help circumvent this potential issue.

Despite showing a significant benefit in LAFS m-mode, this study did not show a significant change in LA size, which was shown in a previous study of apparently healthy cats (20). This discrepancy may be due to differences in duration of pimobendan administration, with the cats in the aforementioned study receiving pimobendan for 7 days vs. single-dose administration in the present study (20). Alternatively, myocardial stiffening and diastolic dysfunction present in HCM cats may prevent the degree of LA size reduction that may be possible in normal cats with normal LV compliance. This same study did not show increases in LAFS, unlike our study (20).

Another LA function measure, auricular flow velocity, was not significantly different between treatment groups in this study. This is consistent with prior research in normal cats, suggesting that although pimobendan increases the degree of LA contraction, it may not affect the speed of LA contraction (20). However, it is possible that pimobendan may have effects on auricular flow velocity in cats with significant LA enlargement and remodeling or the setting of preexisting reduced auricular flow, which was not present in this study population and should be evaluated in future studies.

This study did not show evidence of exacerbation of LVOTO with pimobendan, which has been a major theoretical concern in administration of this drug to cats. There was no significant difference in LVOT velocities between the treatment groups. However, given individual anatomic variation in HCM disease manifestation, consideration of individual cats is prudent. There was one cat that developed SAM after pimobendan administration; this cat received pimobendan on the second study evaluation day. Thus, an increase in sympathetic tone associated with the second day of handling cannot be distinguished from a pimobendan drug effect. Additionally, one cat developed mid LVOTO following pimobendan administration, with LVOT velocity increasing from 0.82 m/s up to 3.25 m/s despite receiving pimobendan on the first examination. However, it is important to note that this cat's HR was higher at the peak LVOT velocity after pimobendan (294 bpm) than after placebo (232 bpm), which may demonstrate more of a HR-dependent effect on LVOTO rather than a direct pimobendan drug effect. This is corroborated by the observable dynamic nature of SAM and LVOTO in cats. Finally, one cat experienced a suspected pre-syncopal episode during the examination. This cat had SAM and mid LVOTO, but the LVOT velocity was only mildly increased following pimobendan (4.55 vs. 4.05 m/s). A direct drug effect again cannot be definitively determined, as sympathetic surge during the examination could also have contributed to the pre-syncopal event, given the patient was particularly fractious. Further studies evaluating other cardiovascular effects of pimobendan, such as blood pressure, in HCM cats are necessary.

Similar to a previous study, we did not identify any observable increase in systolic function at our single time point evaluation (21). In order to identify any impact on systolic function, the prior study averaged effects over multiple evaluations 12 h post-pimobendan administration (20). In the present study, only acute effects were evaluated. This leads us to agree with prior research that the systolic function effects of pimobendan, particularly in the acute setting are either clinically irrelevant or non-existent. The lack of identifiable changes may be due to the presence of HCM pathology in conjunction with marked sympathetic tone of the cats in this study. The cats likely reached a physiologic maximum in systolic function, thus limiting the ability to identify differences between treatment groups; in addition, several cats experienced end-systolic cavity obliteration, which may have contributed to measurement error. This is a challenge inherent to feline research. Future studies may aim to enroll study subjects amenable to handling so as to limit confounding effects of elevated sympathetic tone.

Diastolic function assessment was largely precluded by persistent tachycardia in the cats. Thus, the diastolic function benefits previously demonstrated in dogs and humans could not be explored in these cats (16, 17). Future studies with larger sample sizes or invasive assessments of LV pressure volume relationships may help elucidate diastolic function effects of pimobendan in cats.

The study was limited by the fact that the time to peak physiologic drug effects of pimobendan in cats remains unknown (21). Although the time to peak plasma concentration has been identified and was used in this study as a surrogate for peak physiologic effect, the peak plasma concentration does not necessarily correlate with peak drug effects. Studies involving more long-term administration of pimobendan may help to better characterize pharmacodynamics of the drug in cats with HCM. Additionally, the persistent tachycardia and high sympathetic tone of the study population likely limited the precision of echocardiographic measurements, making it difficult to identify small differences between treatment groups given the small sample size. Another limitation lies in imperfections of spectral Doppler studies since accurate measurement of LVOT velocity requires parallel alignment with the fastest blood flow stream. Although the outflow tract was scanned from multiple angles, with only the fastest flow velocity recorded, it is possible that some velocities are underestimated due to suboptimal alignment. Although the plane of sedation was minimal and sedation may be necessary in clinical feline patients, this could have minimally impacted cardiac function and our results. Finally, the scope of echocardiographic measures assessed in this study were somewhat limited and more advanced echocardiographic assessment of cardiac function or perhaps invasive hemodynamic assessment of cardiac function could represent future study opportunities.

In conclusion, the present study suggests that pimobendan imparts a significant benefit in LA function in cats with HCM and provides evidence that pimobendan does not significantly increase systolic function and LVOT obstructions. The drug appears to be well-tolerated in this group of cats, although the pre-syncopal event experienced by one cat after pimobendan cannot be ruled out as an adverse event directly related to pimobendan. Further studies with longer-term administration of pimobendan to unsedated, compliant cats are indicated to better characterize its safety and physiologic effects and translate these effects to clinical applications.

## Author Contributions

MO, SH, and JS conceived study design. MO, YU, EO, SF, and JS carried out experiments. MO and JS generated and analyzed data. MO wrote initial manuscript draft. MO, YU, EO, SF, SH, and JS edited manuscript draft.

### Conflict of Interest Statement

The authors declare that the research was conducted in the absence of any commercial or financial relationships that could be construed as a potential conflict of interest.

## Abbreviations

CHF: Congestive heart failure
HCM: Hypertrophic cardiomyopathy
HR: Heart Rate
DRVOTO: Right ventricular outflow tract obstruction
LA: Left atrial
LAFS: Left atrial fractional shortening
LV: Left ventricle
LVOT: Left ventricular outflow tract
LVOTO: Left ventricular outflow tract obstruction
M-Mode: Motion-mode echocardiography
RV: Right ventricle
RVOT: Right ventricular outflow tract
SAM: Systolic anterior motion
2D: Two-dimensional echocardiography.
